# Global Screening of Genomic and Transcriptomic Factors Associated with Phenotype Differences between Multidrug-Resistant and -Susceptible Candida haemulonii Strains

**DOI:** 10.1128/mSystems.00459-19

**Published:** 2019-12-17

**Authors:** Hao Zhang, Yifei Niu, Jingwen Tan, Weixia Liu, Ming-an Sun, Ence Yang, Qian Wang, Ruoyu Li, Yejun Wang, Wei Liu

**Affiliations:** a Department of Dermatology and Venereology, Peking University First Hospital, Beijing, China; b Department of Cell Biology and Genetics, Shenzhen University Health Science Center, Shenzhen, China; c Epigenomics and Computational Biology Lab, Biocomplexity Institute of Virginia Tech, Blacksburg, Virginia, USA; d Department of Microbiology & Infectious Disease Center, School of Basic Medical Sciences, Peking University Health Science Center, Beijing, China; e Research Center for Medical Mycology, Peking University, Beijing, China; f National Clinical Research Center for Skin and Immune Diseases, Beijing, China; g Beijing Key Laboratory of Molecular Diagnosis on Dermatoses, Beijing, China; Marquette University

**Keywords:** *Candida haemulonii* complex, comparative genomics, genome, multidrug resistance, transcriptome

## Abstract

Candida haemulonii, a close relative of Candida auris, is an emerging pathogen which frequently shows multidrug resistance especially to triazoles, the most used antifungal drugs. The mechanisms of drug resistance in C. haemulonii, however, are largely unknown. Here, we sequenced and annotated the genomes of two reference strains from the C. haemulonii complex, compared the phenotypes, genomes, and transcriptomes of a triazole-susceptible and two triazole-resistant C. haemulonii strains, and identified triazole susceptibility, morphology, fitness, and the major genetic and gene expression differences between the strains. A multidrug efflux gene, *CDR1*, was recurrently found to be upregulated for expression in triazole-resistant strains. Blocking the activity of Cdr1 increased the susceptibility to triazoles strikingly. Comparative transcriptome analysis also demonstrated impaired cell wall integrity, filamentous growth, and iron homeostasis in triazole-resistant strains. Finally, we also identified a zinc-binding MHR family transcription regulator gene that mutated in triazole-resistant strains spontaneously, contributing to the changes of morphology and, possibly, cell wall integrity between the strains. The study provided important clues for future studies exploring the mechanisms of multidrug resistance and related phenotypic differences of C. haemulonii strains.

**IMPORTANCE** A comprehensive, multi-omic survey was performed to disclose the genetic backgrounds and differences between multidrug-resistant and -susceptible C. haemulonii strains. Genes were identified with mutations or significant expression differences in multidrug-resistant compared to multidrug-susceptible strains, which were mainly involved in multidrug resistance, stress fitness, and morphology. The Cdr1-encoding gene, C. haemulonii *2486* (*CH_2486*), was expressed at a significantly increased level in multidrug-resistant strains. Functional inhibition experiments further implicated potential roles of *CH_2486* in drug resistance. A gene spontaneously mutated in resistant strains, *CH_4347*, was experimentally validated to influence the morphology of spores, possibly by controlling cell wall integrity.

## Abstract (precis)

A comprehensive, multi-omic survey was performed to disclose the genetic backgrounds and differences between multidrug-resistant and -susceptible C. haemulonii strains. Genes were identified with mutations or significant expression differences in multidrug-resistant compared to multidrug-susceptible strains, which were mainly involved in multidrug resistance, stress fitness, and morphology. The Cdr1-encoding gene, C. haemulonii *2486* (*CH_2486*), was expressed at a significantly increased level in multidrug-resistant strains. Functional inhibition experiments further implicated potential roles of *CH_2486* in drug resistance. A gene spontaneously mutated in resistant strains, *CH_4347*, was experimentally validated to influence the morphology of spores, possibly by controlling cell wall integrity.

## INTRODUCTION

In health care settings, the incidence of infections with unusual fungal pathogens has risen tremendously due to the increased morbidity of immune deficiency and the advance of transplant surgery (1–3). Meanwhile, the multidrug resistance of these pathogens has become a severe threat to human (2, 4, 5). In recent years, the proportion of patients with invasive candidiasis caused by Candida haemulonii, a rare non-*albicans Candida* species, has been increasing. The C. haemulonii complex species were originally isolated at different times from blood samples from a patient with renal failure in 1984 (6), and related species were classified into two major groups, namely, C. haemulonii group I (C. haemulonii *sensu stricto* and C. haemulonii var. *vulnera*) and group II (Candida duobushaemulonii) according to isoenzyme profiles, DNA reassociations, physiological characteristics, and phylogenetic analysis (7–9). C. haemulonii, characterized as a possible emerging pathogen, has been reported to be intrinsically resistant to amphotericin B (AMB) and to exhibit *in vitro* multiresistance to triazoles (10–12). These isolates, with a fluconazole (FLC) MIC of ≥64 mg/liter, a voriconazole (VRC) MIC of ≥16 mg/liter, a itraconazole (ITA) MIC of ≥1 mg/liter, or a posaconazole (POS) MIC of ≥2 mg/liter, were defined as triazole-resistant strains based on *in vitro* antifungal susceptibility data (10, 11). In recent years, clinical infections by Candida auris, a phylogenetic relative of C. haemulonii, have been frequently reported worldwide (13–17). C. auris, the so-called “superbug fungus,” also shows *in vitro* multidrug resistance to triazoles, AMB, and echinocandins (18). The Centers for Disease Control and Prevention have continually updated the epidemiology and showed special concern regarding this fungus, since it often shows multidrug resistance, is difficult to identify, and causes outbreaks in health care settings (18–21). C. haemulonii has received less attention than C. auris. However, it also causes outbreaks, shows multidrug resistance, and leads to high mortality (2, 12).

Triazoles are the most widely used antifungal agents, targeting lanosterol 14-α-demethylase (Erg11) and inhibiting biosynthesis of membrane ergosterol (22, 23). In general, triazole resistance is common in various pathogenic fungi clinically, the mechanisms of which in fungi involve mutation or overexpression of Erg11, upregulation of multidrug transporters or cellular stress responses, etc. (23). For example, recent studies suggested that mutation in *ERG11* in C. auris could confer triazole resistance (24). Genetic analysis of C. auris also implicated the contribution of upregulated expression of Cdr1, a multidrug efflux pump, to the triazole resistance (25). However, the exact mechanisms of triazole resistance in C. haemulonii remain to be investigated, although previous studies suggested possible roles of transporters and metabolic regulators in drug responses (26).

In this work, we investigated four strains of C. haemulonii complex; one was a triazole-susceptible C. haemulonii strain (BMU05228), and the rest were triazole-resistant strains, including one C. duobushaemulonii strain (BMU05314) and two C. haemulonii strains (BMU05529 and BMU05535). BMU05529 and BMU05535 were recovered from a single patient at different times. BMU05228 and BMU05314 were selected as the representative strains and sequenced for the genomes with both second-generation and single-molecule sequencing platforms. Genome resequencing and RNA sequencing were also performed for the two multidrug-resistant C. haemulonii strains. Comparisons were performed between the multidrug-resistant and -susceptible strains to screen the genetic and gene expression differences, followed by experimental validation of the interesting targets which might explain the mechanisms of triazole resistance and phenotypic changes in C. haemulonii strains.

## RESULTS

### Identification and phenotypes of four C. haemulonii complex strains.

**FIG 1:**
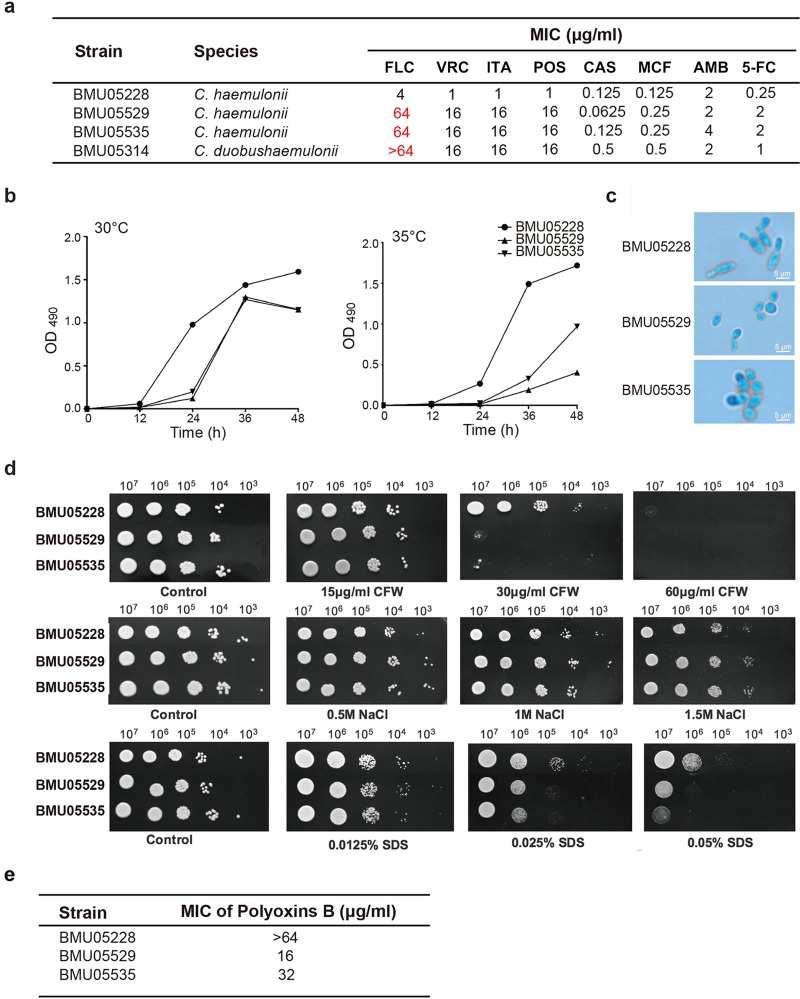
The phenotype differences among C. haemulonii isolates. (a) Susceptibility of C. haemulonii and C. duobushaemulonii isolates to different antifungal drugs. (b) Growth curves under conditions of different temperatures. A total of 1 × 10^3^ C. haemulonii cells were diluted with 100 μl 1640 medium and inoculated into 96-well plates. The growth speeds (represented by values of OD_490_) at 30°C and 35°C were examined at different time points through XTT assay. Average OD_490_ values determined from three repeats at every time point are presented. (c) Cell morphology under a light microscope (×400 magnification). (d) Fitness under conditions of exposure to environmental stress. C. haemulonii cells were adjusted to 5 × 10^8^ cells/ml, and then 10-fold serial dilutions of cells (2 μl) were spotted on YPD plates and on YPD plates containing CFW, NaCl, or SDS at different concentrations for 2 days of growth. (e) Susceptibility to polyoxin B of C. haemulonii isolates. Abbreviations: FLC, fluconazole; VRC, voriconazole; ITA, itraconazole; POS, posaconazole; CAS, caspofungin; MCF, micafungin; AMB, amphotericin B; 5-FC, 5-flucytosine; CFW, calcofluor white.

We isolated four fungal strains from infected patients with candidemia. All the strains were identified as C. haemulonii complex by amplifying and sequencing internal transcribed spacer 1 (*ITS1*) and *ITS4* and *D1*/*D2*, with three (BMU05228, BMU05529, and BMU05535) being identified as C. haemulonii *sensu stricto* and one (BMU05314) as C. duobushaemulonii, exhibiting 100% sequence identity to the corresponding sequences from reference C. haemulonii complex strains (C. haemulonii *sensu stricto* CBS 5149 and C. duobushaemulonii CBS 7798) (Fig. 1a). The broth microdilution method was used to test antifungal susceptibility of these strains, and the phenotype (susceptibility or resistance) was determined as described in previous reports (10, 11). As shown in Fig. 1a, BMU05228 was susceptible to FLC while the remaining strains were all resistant. BMU05228 also appeared to be more susceptible than BMU05529, BMU05535, and BMU05314 to other triazoles, such as VRC and POS, but no differences were observed in susceptibility to other antifungal agents (Fig. 1a).

Other phenotypes were also compared among BMU05228, BMU05529, and BMU05535 since the three strains belonged to the same species whereas they showed different antifungal resistance profile. At both 30°C and 35°C, BMU05228 grew more efficiently than BMU05529 and BMU05535, with a more striking advantage seen as the temperature increased (Fig. 1b). In contrast to BMU05228, for which the spores appeared with a rod-like morphology and a median size of 2.4 by 7.0 μm, BMU05529 and BMU05535 cells showed a round shape with a median size of 3.0 by 4.0 μm (Fig. 1c). The levels of fitness with respect to environmental stimuli were also different between the strains. BMU05228 showed better fitness than BMU05529 and BMU05535 under conditions of stimulation with calcofluor white (CFW) (30 μg/ml) and SDS (0.025%), but no differences were seen with respect to responses to salt exposure (Fig. 1d). CFW is involved in the biosynthesis of fungal cell wall. When polyoxin B, an inhibitor of chitin synthase, was applied, BMU05529 and BMU05535 were more susceptible than BMU05228, further suggesting that the reduced fitness of the triazole-resistant strains might be caused by impairment of the cell wall (Fig. 1e).

### Genomic mutation patterns in triazole-resistant C. haemulonii strains.

To further screen the candidate genes responsible for the differences in drug resistance and other phenotypes in C. haemulonii complex, the genomes of representative strains were sequenced. We assembled and annotated high-quality genomes for triazole-susceptible C. haemulonii and C. duobushaemulonii, respectively, followed by resequencing the genomes of other C. haemulonii strains and comparing the genomes.

Triazole-susceptible C. haemulonii strain BMU05228 and the only C. duobushaemulonii strain, BMU05314, were selected as representative strains for reference genome sequencing and annotation (Materials and Methods) (see also Table S1 and S2 in the supplemental material for a summary of the sequencing and annotation methods). The reference genomes were 13,305,295 nucleotides (nt) and 12,568,244 nt in size, respectively, for BMU05228 and BMU05314 (Table S1). High levels of sequence similarity and conserved genome synteny were found between BMU05228 and BMU05314 and the genomes of a C. haemulonii strain and a C. duobushaemulonii strain recently reported by Munoz et al., respectively (26) (see Fig. S1 in the supplemental material). Transcriptome sequencing (RNA-Seq) facilitated the identification of 6,155 and 5,943 genes in BMU05228 and BMU05314, respectively, among which 5,964 and 5,761 genes encoded proteins (Table S2). The distribution of exon numbers per gene and protein family types and gene function annotation were analyzed, and the results are shown in Fig. S2a to d and Table S2. The core gene set was also retrieved from representative strains of *Ascomycota*, followed by construction of a core genome tree. The tree showed a robust topology consistent with the evolutionary relationships disclosed by traditional taxonomy (Fig. S3a), and the data were further confirmed by the trend of changes for pairwise protein similarities of core genes (Fig. S3b) and the numbers of homologs or orthologs between BMU05228 and the other strains (Fig. S3c). Comparative genomic analysis was also performed between BMU05228 and BMU05314 and the other representative *Ascomycota* strains. We identified a list of unique genes of BMU05228 and BMU05314 that were not identified with homologs in other strains. Among them, 81 C. haemulonii genes showed homology with 71 C. duobushaemulonii counterparts and therefore were C. haemulonii complex specific; we were able to classify those genes as 50 singletons and members of 10 multimember families (Fig. S3d; see also Data Set S1 in the supplemental material). The remaining 651 C. haemulonii and 674 C. duobushaemulonii genes were species/strain specific, and all of them were classified as singletons (Fig. S3d; see also Data Set S1).

10.1128/mSystems.00459-19.1 FIG S1 Collinearity and synteny between BMU05228 and GCF_002926055.1 genome assemblies (a and b) and between BMU05314 and GCF_002926085.1 genome assemblies (c and d). Download FIG S1, TIF file, 1.6 MB. Copyright © 2019 Zhang et al. 2019 Zhang et al. This content is distributed under the terms of the Creative Commons Attribution 4.0 International license.

10.1128/mSystems.00459-19.2 FIG S2 Gene annotation of the representative C. haemulonii and C. duobushaemulonii strains. (a and b) Distribution of exons (a) and protein families (b) in BMU05228 and BMU05314 strains. (c) Function annotation of the genes by comparison with external databases. (d) Virulence gene annotation. Download FIG S2, TIF file, 1.6 MB. Copyright © 2019 Zhang et al. 2019 Zhang et al. This content is distributed under the terms of the Creative Commons Attribution 4.0 International license.

10.1128/mSystems.00459-19.3 FIG S3 Comparison of BMU05228 and BMU05314 genomes to those of close relatives. (a) Neighbor-joining phylogenomic tree based on the *Ascomycota* core genomes. (b) Homology between the protein ortholog pairs in BMU05228 and other strains. (c) The number of homolog and orthologs between BMU05228 and other strains. (d) Protein family types and their distributions in and between BMU05228 and BMU05314. Download FIG S3, TIF file, 1.1 MB. Copyright © 2019 Zhang et al. 2019 Zhang et al. This content is distributed under the terms of the Creative Commons Attribution 4.0 International license.

10.1128/mSystems.00459-19.6 TABLE S1 Summary of genome sequencing and assemblies of C. haemulonii BMU05228 and C. duobushaemulonii BMU05314. Download Table S1, DOCX file, 0.06 MB. Copyright © 2019 Zhang et al. 2019 Zhang et al. This content is distributed under the terms of the Creative Commons Attribution 4.0 International license.

10.1128/mSystems.00459-19.7 TABLE S2 Genome summary and gene annotation of C. haemulonii strain BMU05228 and C. duobushaemulonii strain BMU05314. Download Table S2, DOCX file, 0.05 MB. Copyright © 2019 Zhang et al. 2019 Zhang et al. This content is distributed under the terms of the Creative Commons Attribution 4.0 International license.

10.1128/mSystems.00459-19.8 DATA SET S1 C. haemulonii complex-specific genes. Download Data Set S1, XLSX file, 0.2 MB. Copyright © 2019 Zhang et al. 2019 Zhang et al. This content is distributed under the terms of the Creative Commons Attribution 4.0 International license.

**FIG 2:**
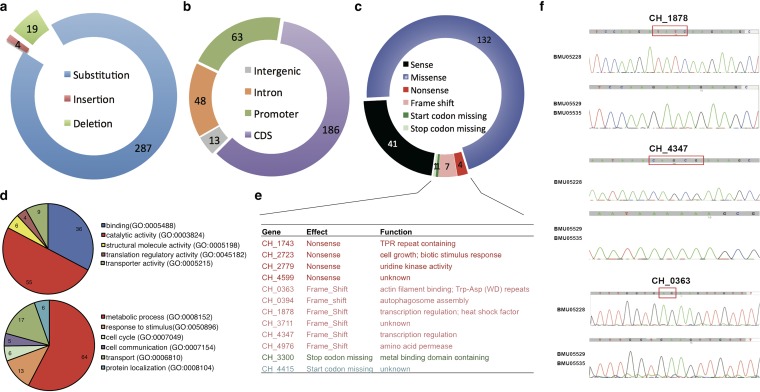
Single nucleotide polymorphisms or small insertion/deletions in triazole-resistant C. haemulonii strains. (a) Composition of genomic mutations. (b) Distribution of regions of genomic mutations. CDS, coding DNA sequence. (c) Effect of the genomic mutations within protein-encoding regions. (d) Molecular function and biological processes of the proteins with nucleotide mutations. (e) Annotation of the genes with nonsense, frame shifting, and mutations with start or stop codon missing. TPR, tetratricopeptide repeat. (f) Verification of the mutations by gene resequencing.

Furthermore, we resequenced the genomes of BMU05529 and BMU05535, the two triazole-resistant C. haemulonii strains. Alignment against the BMU05228 reference genome disclosed 23 small insertions/deletions and 287 single nucleotide substitutions (Fig. 2a; see also Data Set S2). An absolute majority of the single nucleotide polymorphisms (SNPs) was consistently called from both the two triazole-resistant strains, with only a few identified from one strain but not from the other due to the regional low read coverage (Data Set S2). The only discrepancy with respect to different SNP patterns in the two strains was located in C. haemulonii *3711* (*CH_3711*) with a binucleotide insertion in BMU05529 but a deletion in the same position of the same two nucleotides in BMU05535, both leading to the gene frameshift (Data Set S2).

10.1128/mSystems.00459-19.9 DATA SET S2 SNPs between FLC-susceptible and -resistant C. haemulonii strains. Download Data Set S2, XLSX file, 0.07 MB. Copyright © 2019 Zhang et al. 2019 Zhang et al. This content is distributed under the terms of the Creative Commons Attribution 4.0 International license.

Genome localization of the 310 mutations indicated that 186 were located in exons encoding peptide sequences, 63 in proximity of encoding genes and possible promoter regions, 48 within introns, and 13 in intergenic regions (Fig. 2b). Among the mutations seen in protein-encoding frames, 132 caused amino acid changes (missense), 7 caused frame shifts, 4 caused premature translation termination (nonsense), and 2 led to loss of translation start or termination codons (Fig. 2c). Functional clustering was performed on the genes with mutations causing frame shifts, amino acid changes, premature translation termination, and loss of translation start or stop codons (Fig. 2d). It was noted that, in addition to metabolism-related genes, genes with transporter activity and participating in response to stimulus were also enriched (Fig. 2d). The genes with mutations causing frame changes were listed and annotated for their possible function (Fig. 2e). Two of them, *CH_1878* and *CH_4347*, were annotated as transcription regulators. Independent cloning and sequencing of these two genes and of another one possibly encoding an actin filament binding protein (*CH_0363*) in the two triazole-resistant strains further confirmed the frame-shifting mutations (Fig. 2f).

### Constitutive gene expression differences between triazole-susceptible and -resistant C. haemulonii strains.

**FIG 3:**
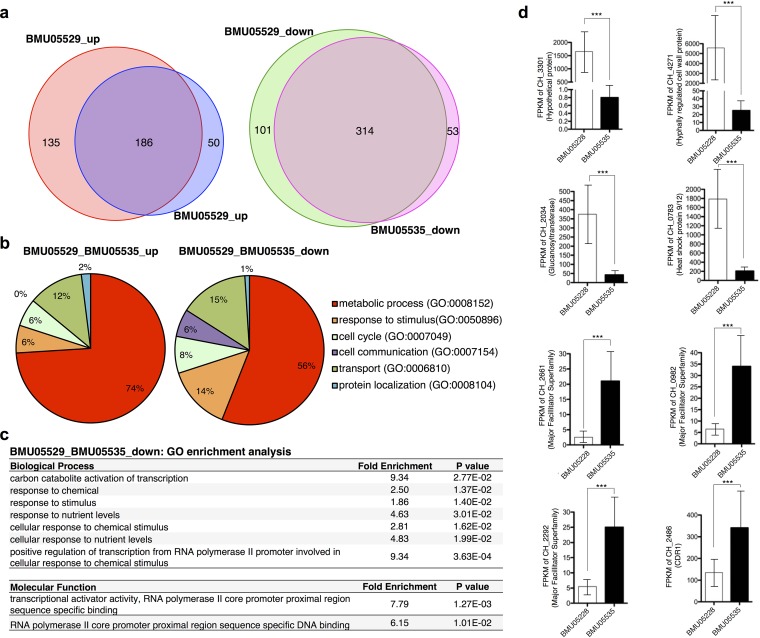
Gene expression differences between triazole-susceptible and -resistant C. haemulonii strains without drug treatment. (a) Genes with significant expression differences between BMU05228 and BMU05229 or between BMU05228 and BMU05535. (b) Biological processes of the genes with significant expression differences. (c) GO enrichment analysis for the genes downregulated in triazole-resistant strains. (d) Expression of selected genes with significant differences. FPKM, fragments per kilobase per million (1,000-bp and 1-million-bp reads). ***, false-discovery rate (*q*) < 0.005 (Cufflinks [cuffdiff] transcript expression comparison).

RNA sequencing was also performed to observe differences in the gene expression profiles of triazole-susceptible and -resistant C. haemulonii strains incubated without any drug treatment. The differentially expressed genes were extensively consistent between the two triazole-resistant strains compared to the triazole-susceptible one, with 186 and 314 protein-encoding genes in total for which expression was commonly up- and downregulated in the triazole-resistant strains, respectively (Fig. 3a; see also Data Set S3). Among the differentially expressed genes, 35 were also found with genomic mutations between triazole-susceptible and -resistant C. haemulonii strains (Data Set S3). Interestingly, 11 mutations were located in promoter regions, which might represent direct associations with the gene expression differences (Data Set S3).

10.1128/mSystems.00459-19.10 DATA SET S3 Genes with different expression levels and mutations between FLC-susceptible and -resistant C. haemulonii strains with and without FLC treatment. Download Data Set S3, XLSX file, 1.3 MB. Copyright © 2019 Zhang et al. 2019 Zhang et al. This content is distributed under the terms of the Creative Commons Attribution 4.0 International license.

Consistent with the mutation profiling results, functional clustering of the genes with significant expression changes also revealed that the majority of genes were involved in transport or in responses to stimulus, besides metabolic processes (Fig. 3b). Interestingly, the functional composition of genes with downregulated expression in triazole-resistant strains appeared very similar to that of genes with meaningful mutations in the same strains (Fig. 3b; see also Fig. 2d, lower panel). GO enrichment analysis further indicated that the genes that participated in stimulus response or in transcriptional regulation were significantly enriched in the sets of downregulated genes of triazole-resistant strains (Fig. 3c). Expression data are shown for representative genes with significant differences in levels of expression between triazole-resistant and -susceptible strains (Fig. 3d). It was noted that, among the genes most significantly downregulated in the triazole-resistant strains, there were many involved in biosynthesis or in maintenance of the cell wall, e.g., *CH_4271* and *CH_2034* (Fig. 3d). In contrast, the genes most significantly upregulated in the triazole-resistant strains were often involved in transport or multidrug resistance. For example, *CH_2486* encoded Cdr1, a protein widely reported to be associated with antifungal resistance, especially resistance to triazoles (Fig. 3d). *CH_2661*, *CH_0892*, and *CH_2292* were other examples of genes encoding transporters that might play roles in drug resistance (Fig. 3d).

### FLC-induced gene expression differences between triazole-susceptible and -resistant C. haemulonii strains.

**FIG 4:**
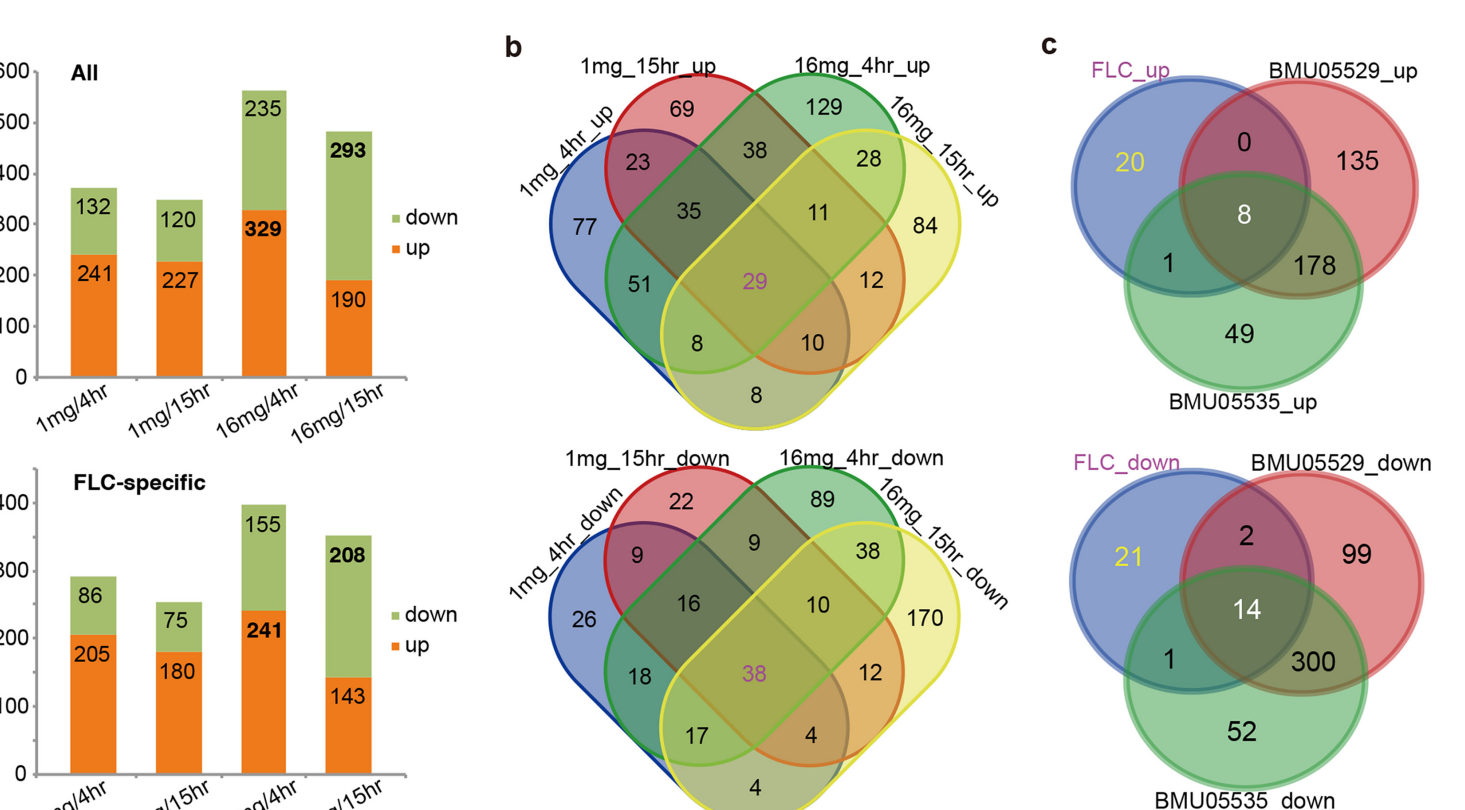
Gene expression differences between triazole-susceptible and -resistant C. haemulonii strains with or without FLC treatment. (a) All the genes with significant expression differences between triazole-susceptible and -resistant strains treated with different doses of FLC for different durations. The numbers of genes down- or upregulated in triazole-resistant strains are shown. (b) Specific or common genes with differential expression levels among various FLC treatment regimen groups. (c) The specific or common genes with differential expression levels among FLC treatment and no-drug treatment groups.

To further observe the differences between the triazole-susceptible and -resistant strains in transcriptomic responses to drug stimulation, we also performed RNA-Seq analysis on the strains under conditions of incubation with 1 mg/liter and 16 mg/liter FLC for 4 h and 15 h, respectively. FLC used at 16 mg/liter stimulated strikingly more genes with different responses between triazole-resistant and -susceptible strains, with the greatest number of upregulated genes seen at 4 h and the greatest number of downregulated genes at 15 h in the triazole-resistant strains (Fig. 4a; see also Data Set S3). There were 29 and 38 genes that consistently showed significantly increased and decreased expression in the triazole-resistant strains, respectively (Fig. 4b). Around 28% (8/29) of the upregulated genes and 37% (14/38) of the downregulated genes showed stable expression differences between the strains with or without drug stimulation, while most genes (20/29 upregulated and 21/38 downregulated) appeared to respond to FLC stimulation specifically since they were not covered by the list of gene showing differential levels of expression between the strains without FLC treatment (Fig. 4c). Functional annotation of the constantly upregulated genes in triazole-resistant strains identified two genes (*CH_2486* and *CH_4618*) as being directly associated with drug resistance (Table 1). Both the genes encode transporters, and, as indicated before, *CH_2486* encodes Cdr1, which is well known to exhibit triazole-resistant activity in fungi by removing drugs from cells. Two genes showed cytochrome or oxidation-reduction activity that could be related to cellular responses to drug stresses. Among the constantly downregulated genes in triazole-resistant strains, genes were identified that were remarkably enriched for participating in cell wall biosynthesis or maintenance, filamentous growth, iron ion transport and assimilation, and other phenotypic switching processes (Table 1). It should be noted that the functions of the enriched genes were frequently correlated. We also annotated the function of genes that specifically showed responses to FLC stimulation (Table 2). Similarly, metabolism-related genes and oxidation-reduction reaction-related genes responding to drug were enriched among those listed as upregulated genes in triazole-resistant strains, while genes associated with cell wall organization, filamentous growth, and iron homeostasis were enriched in the downregulated gene list (Table 2). Together, the results suggested increased activities for multidrug efflux and stress-reactive metabolism and decreased activities or capabilities for cell wall organization, filamentous growth, iron assimilation, and pathogenesis.

**TABLE 1:** Functional annotation for the genes with constitutive and constant expression differences between triazole-susceptible and -resistant strains

Gene	Function
Upregulated in triazole-resistant strains	
*CH_2486*	Azole transmembrane transporter
*CH_4618*	ABC-type multidrug transporter
*CH_5962*	Cytochrome activity
*CH_4764*	NAD(P)+ activity
*CH_5380*	Biofilm formation
*CH_2661*	Iron ion transport
*CH_5642*	Isopenicillin N synthase
*CH_2221*	Uncharacterized
	
Downregulated in triazole-resistant strains	
*CH_4271*	Cell wall organization
*CH_5611*	Cell wall organization
*CH_4284*	Filamentous growth
*CH_4269*	Filamentous growth
*CH_3050*	Filamentous growth
*CH_3038*	Filamentous growth; TF
*CH_4640*	Regulation of phenotypic switching
*CH_2686*	Regulation of phenotypic switching
*CH_1562*	Iron ion homeostasis
*CH_5586*	Iron ion homeostasis
*CH_1713*	Iron ion homeostasis
*CH_2161*	Uncharacterized
*CH_4008*	Uncharacterized
*CH_3052*	Uncharacterized

**TABLE 2:** Functional annotation for the genes with expression differences induced by fluconazole treatment between triazole-susceptible and -resistant strains

Gene	Function
Upregulated in triazole-resistant strains	
*CH_3590*	Ribosome biosynthesis; response to drug
*CH_5190*	Ribosome biosynthesis; response to drug
*CH_1406*	Ribosome biosynthesis; response to drug
*CH_5963*	Ribosome biosynthesis; response to drug
*CH_3374*	Ribosome biosynthesis; response to drug
*CH_2488*	U2-type prespliceosome
*CH_2511*	U2-type spliceosomal complex
*CH_4406*	Transcriptional activator
*CH_1721*	Proteolysis in the vacuole
*CH_5641*	Glucan catabolic process
*CH_2296*	Cytochrome activity
*CH_0186*	NAD(P)+ activity
*CH_4837*	NAD activity
*CH_3201*	NADPH activity
*CH_1275*	Uncharacterized
*CH_4930*	Uncharacterized
*CH_4393*	Uncharacterized
*CH_3192*	Uncharacterized
*CH_0070*	Uncharacterized
*CH_5314*	Uncharacterized

Downregulated in triazole-resistant strains	
*CH_3203*	Cell wall organization
*CH_1703*	Cell wall organization; iron ion homeostasis
*CH_4297*	Cell wall organization; iron ion homeostasis
*CH_4857*	Iron ion homeostasis
*CH_4342*	Iron ion homeostasis
*CH_4295*	Iron ion homeostasis
*CH_4292*	Iron ion homeostasis
*CH_5516*	Filamentous growth
*CH_3010*	Filamentous growth
*CH_0317*	Fucose/proton symporter activity
*CH_3049*	Rab guanyl-nucleotide exchange
*CH_5515*	Uncharacterized
*CH_2129*	Uncharacterized
*CH_4309*	Uncharacterized
*CH_4310*	Uncharacterized
*CH_4294*	Uncharacterized
*CH_4270*	Uncharacterized
*CH_3524*	Uncharacterized
*CH_0003*	Uncharacterized
*CH_0374*	Uncharacterized
*CH_3918*	Uncharacterized

### *CH_2486* expression is likely associated with triazole resistance of the C. haemulonii strains.

**FIG 5:**
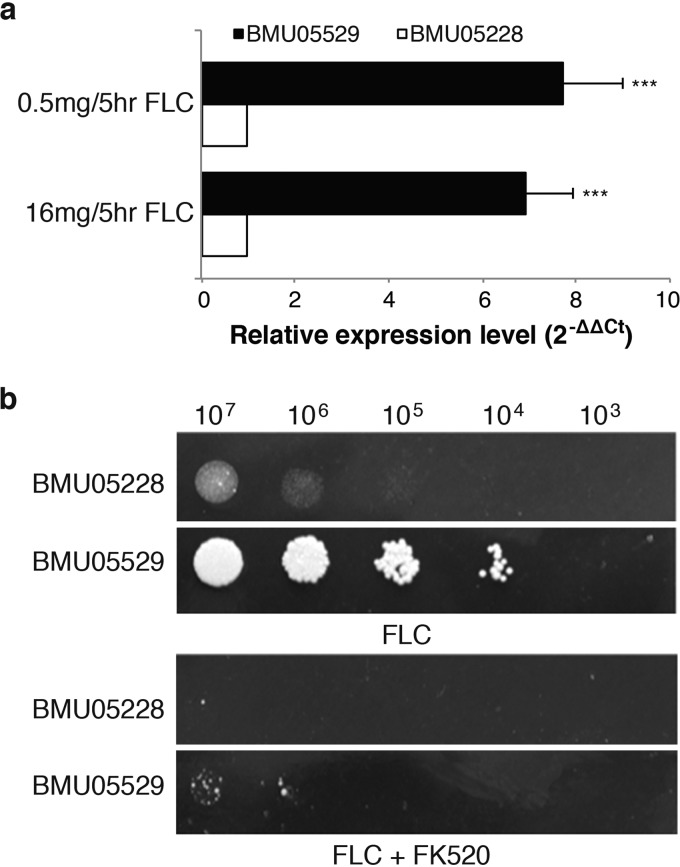
The expression level of efflux pump (Cdr1) was increased in the triazole-resistant C. haemulonii strain. (a) qRT-PCR analysis of *CH_2486* gene expression in BMU05228 and BMU05529 treated with different doses of fluconazole (FLC) for 5 h. A total of 1 × 10^6^ C. haemulonii cells were first incubated for 10 h and then treated with 0.5 mg/liter or 16 mg/liter FLC for another 5 h. Cells were harvested, and total RNA was extracted to detect the relative expression levels of *CH_2486* (*CDR1*) through qRT-PCR. The data are shown as means ± standard deviations (*n* = 3). ***, *P* < 0.005 (Student's *t* test for the logarithm value). (b) Susceptibility of BMU05228 and BMU05529 strains to FLC with and without FK520 treatment. C. haemulonii cells were adjusted to 5 × 10^8^ cells/ml, and then 10-fold serial dilutions of cells (2 μl) with or without 100 mg/liter FK520 treatment were spotted on YPD plates containing 64 mg/liter FLC for 2 days of growth.

Transcriptome analysis showed constitutively increased expression of *CH_2486* (*CDR1*) in the triazole-resistant C. haemulonii strains. The higher expression of *CH_2486* in strain BMU05529 than in strain BMU05228 was further validated by real-time quantitative PCR (qRT-PCR) after treatment with different doses of FLC (Fig. 5a). There was only one anonymous nucleotide substitution detected within the protein coding sequences of strain BMU05228 and strains BMU05529 and BMU05535, and no nucleotide changes were found within the gene promoter regions (Data Set S3). Therefore, the *CH_2486* gene expression differences were likely caused by the changes in upstream regulator genes.

**FIG 6:**
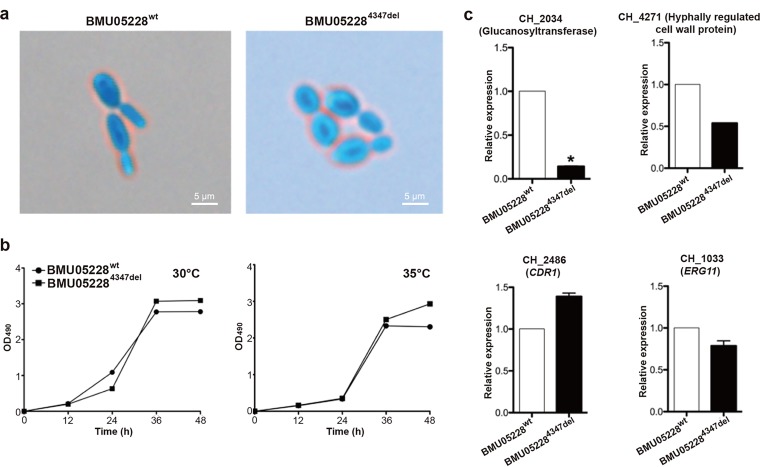
Phenotype and gene expression differences between wild-type and *CH_4347* knockout BMU05228 strains. (a) Morphology differences under light microscope (×400 magnification). (b) Growth curves of the strains under conditions of 30°C and 35°C. Average OD_490_ values of three repeats at every time point were examined through the methods described above. (c) Gene expression determined by qRT-PCR. A total of 1 × 10^6^ C. haemulonii cells were incubated for 15 h. Then, cells were harvested and total RNA was extracted to detect the relative expression levels of genes through qRT-PCR. The data are shown as means ± standard deviations (*n* = 3). *, *P* < 0.05 (Student's *t* test for the logarithm value).

To examine whether the *CH_2486* expression differences changed the susceptibility of C. haemulonii to FLC, we treated the strains with FK520, a putative Cdr1 inhibitor. As shown in Fig. 6b, BMU05529 was much more resistant to FLC than BMU05228. However, after simultaneous incubations with FK520, both strains showed increased susceptibility to FLC, and BMU05529 became as nearly vulnerable as BMU05228 to the same dose of FLC (Fig. 5b). Therefore, *CH_2486* could be an important factor endowing the C. haemulonii strains with a capacity for resistance to FLC and other triazoles.

### *CH_4347* mutation is associated with spore morphology differences in triazole-resistant C. haemulonii strains.

Experiments were also performed to investigate whether other genes screened by genome or RNA sequencing had functional relevance and associations with the observed phenotypic differences of the strains. As annotated, *CH_4347* likely encoded a transcription regulator, and there was a frame-shifting mutation found in the triazole-resistant strains by genome resequencing and validation experiments (Fig. 2e and f). We successfully knocked the gene out from the wild-type BMU05228 strain. Triazole susceptibility tests were performed to compare the *CH_4347*-deleted (BMU05228^4347del^) and wild-type BMU05228 strains. However, no significant difference was observed (Fig. S4). The morphologies of the two strains were also compared, and interestingly, spores of BMU05228^4347del^ showed a round shape similar to that of spores of the triazole-resistant strains, i.e., BMU05529 and BMU05535 (Fig. 6a; see also Fig. 1c). The levels of growth efficiency of BMU05228^4347del^ and the wild-type BMU05228 strain were more similar than those measured for the wild-type BMU05529 and BMU05535 strains (Fig. 6b; see also Fig. 1b). Therefore, *CH_4347* is likely the key gene responsible for the morphology of the C. haemulonii complex spores.

10.1128/mSystems.00459-19.4 FIG S4 Susceptibility to triazoles of the BMU05228 strain and *CH_4347* mutant. (Top) Disk diffusion assay. A total of 1 × 10^5^ cells of each isolate were spread on a YPD plate. Blank paper disks (8 mm in diameter) were impregnated with fluconazole (FLC), voriconazole (VRC), itraconazole (ITA), or posaconazole (POS). Dried disks were placed at the center of inoculated agar plates. The plates were incubated at 30°C in dark for 48 h, and the plates were photographed. (Bottom) Susceptibility to triazoles of the BMU05228 strain and the *CH_4347* mutant was determined through the use of a CLSI broth microdilution method. Download FIG S4, TIF file, 2.6 MB. Copyright © 2019 Zhang et al. 2019 Zhang et al. This content is distributed under the terms of the Creative Commons Attribution 4.0 International license.

Quantitative expression analysis was performed to observe the possible gene expression differences between BMU05228^4347del^ and wild-type strains. One gene involved in cell wall biosynthesis, *CH_2034*, was found with a significantly decreased expression level in BMU05228^4347del^, the results thus being similar to those seen with the triazole-resistant strains (Fig. 6c). Other genes related to cell wall function, e.g., *CH_4271*, also showed a trend of expression change similar to that seen in the triazole-resistant strains, but the data did not reach statistical significance (Fig. 6c and data not shown). We also compared the expression levels of possibly drug resistance-related genes between wild-type BMU05228 and its *CH_4347* mutant strains, e.g., those with mutations in *CH_2486* (*CDR1*), *CH_1033* (*ERG11*), etc., and no expression differences were observed (Fig. 6c; see also Fig. S5). Therefore, *CH_4347* mutation might not represent an independent factor contributing to the phenotype of drug resistance.

10.1128/mSystems.00459-19.5 FIG S5 qRT-PCR results determined for representative genes in the BMU05228 strain and *CH_4347* mutant. The relative expression levels of genes were examined through the use of the methods described above. Download FIG S5, TIF file, 1.0 MB. Copyright © 2019 Zhang et al. 2019 Zhang et al. This content is distributed under the terms of the Creative Commons Attribution 4.0 International license.

## DISCUSSION

The C. haemulonii complex is a group of emerging fungal pathogens that represent a serious health threat globally. Outbreaks of infection by C. auris, a species closely related to the C. haemulonii complex, have recently caused alarm worldwide (https://www.cdc.gov/fungal/diseases/candidiasis/candida-auris.html). Bursts of C. haemulonii complex infections are mainly attributed to their multidrug resistance and our lack of knowledge concerning these microorganisms. C. haemulonii complex showed resistance to AMB and also frequently to triazoles, leading to failures in clinical treatment (11, 12). The combination of FLC with AMB has also been commonly used in clinical practice, and yet the outcomes have been poor in treatment of deep-tissue infections (2, 12). The multidrug resistance of these fungi has further caused high mortality rates. For example, the mortality rate of C. auris infections was previously reported to reach 60% (19). At present, species of the C. haemulonii complex show high sensitivity to echinocandins, but the high price has severely restricted their application. On the other side, we remain at the very beginning in understanding the biology of these previously “rare” pathogens. Robust methods still lack quick and accurate identification of the strains (27, 28). Misidentifications happen frequently and hamper the effective treatment of the infections (4, 29). The genetic and molecular mechanisms of the multidrug resistance of these fungi remain largely unknown.

C. haemulonii strain BMU05228 was found to be susceptible to triazoles, while the other strains (BMU05529 and BMU05535) showed resistance (Fig. 1a). Genome resequencing of the triazole-resistant strains disclosed 7 frame-shifting, 4 nonsense, 2 translation start/termination-interfering, and 132 missense mutations compared with the reference genome of triazole-susceptible BMU05228 (Fig. 2a to c). Together with the mutations present in potential promoter regions, the influenced genes mainly participated in metabolism, transport, and response to stimulus (Fig. 2d). Which of the mutations are functional remains unknown. However, RNA sequencing analysis also disclosed similar functional enrichment of the influenced genes in the set of downregulated genes of triazole-resistant strains (Fig. 3b and c). The consistency between gene expression profiles and genome mutations could highlight the main different biological processes and pathways between the strains.

RNA sequencing analysis of the strains in the presence or absence of FLC treatment repeatedly identified upregulation of a multidrug transporter gene (*CDR1*) and of genes with responses to drugs in triazole-resistant C. haemulonii strains. The upregulated expression of *CDR1* was further validated by qRT-PCR (Fig. 5a). Treatment of the triazole-resistant C. haemulonii strains with Cdr1 inhibitor restored their susceptibility to FLC at least partially (Fig. 5b), further indicating the significant contribution of Cdr1 to triazole resistance. It remains unclear whether other transporters or drug-responding genes also contribute to the drug resistance for the strains. Previous studies suggested that overexpression of multidrug efflux pump MexGHI-OpmD was capable of causing growth deficiency in Pseudomonas aeruginosa (30), and overexpression of the multidrug efflux pump SmeDEF was shown to impair fitness and decrease virulence in Stenotrophomonas maltophilia (31). In triazole-resistant Candida albicans, overexpression of efflux pump also led to impaired fitness in response to novel stresses in the absence of drug (32, 33). Hence, it would be tentative to hypothesize and investigate experimentally the possibility that the slowed growth and impaired fitness to CFW and SDS observed in the triazole-resistant C. haemulonii strains was related to the increased expression of *CDR1* and other efflux pump genes. It has also been reported that defects in cell wall integrity in C. albicans could decrease the growth rate, promote sensitivity to CFW, and modestly reduce adherence (34). We also observed the typical downregulation of cell wall biosynthesis genes in triazole-resistant C. haemulonii strains (Fig. 4). It would be interesting to further examine the potential links between cell wall integrity and cell morphology, growth, stress fitness, and pathogenesis in C. haemulonii strains. We found increased susceptibility to polyoxin B for triazole-resistant C. haemulonii strains, which could also be attributable to the defect of cell wall integrity, since polyoxin B transport was affected by cell wall integrity (35).

The RNA sequencing results also demonstrated that expression of *CH_2034* encoding glucanosyltransferase was significantly decreased in triazole-resistant C. haemulonii strains (Fig. 3d). Previous studies suggested that glucanosyltransferase was involved in cell wall assembly and morphogenesis as shown by elongation of β-(1,3)-glucan chains in the cell wall during vegetative growth (36, 37). We observed apparent morphological differences between strain BMU05228 and strains BMU05529 and BMU05535, with the former strain forming rod-like cells and the latter strains rounder cells (Fig. 1c). We successfully knocked out one gene (*CH_4347*) in BMU05228, which mutated with frame shifting in triazole-resistant strains spontaneously, and found that that gene contributed to the morphology changes between triazole-susceptible and -resistant C. haemulonii strains (Fig. 6a). *CH_2034* also appeared to show a lower level of expression in the gene knockout cells (Fig. 6c). Hence, *CH_4347* might influence the morphology of spores in C. haemulonii by regulating the expression of *CH_2034*. However, no apparent susceptibility difference was observed between the wild-type and *CH_4347* mutant C. haemulonii strains. More experiments should be performed to examine how *CH_4347* influences morphology and to test whether *CH_4347* and other factors make synergic contributions to antifungal susceptibility or virulence. *CH_4347* encodes a zinc-binding MHR family transcription regulator but shows low homology with genes encoding other proteins with well-annotated function. Zinc-binding MHR family proteins have been previously reported to be involved in various functions in other fungi, including morphogenesis, virulence, drug resistance, etc. (38–40). Heat shock factors (HSFs) were also previously reported to regulate morphogenesis and virulence in C. albicans (41, 42). They were shown to regulate the morphology shifting of C. albicans from yeast to filament, representing a phenotype similar to a change regulated by *CH_4347* in C. haemulonii (41). Interestingly, we also found one HSF gene (*CH_1878*) which showed a loss of protein-encoding capability and function in triazole-resistant C. haemulonii strains (Fig. 2e and f). Experiments to separately and jointly mutate and recover these and other frame-changing genes and to observe whether antifungal susceptibility and other phenotypes are influenced are still ongoing.

In this research, we presented chromosome-level, well-annotated reference genomes for two C. haemulonii complex species. Genome resequencing and RNA sequencing performed for the triazole-resistant C. haemulonii strains screened a list of interesting genes, providing important targets and clues for future studies on exploring the genetic and molecular mechanisms of multidrug resistance in C. haemulonii strains. The data comprise valuable resources for the research and clinical communities with respect to candidiasis.

## MATERIALS AND METHODS

### Isolation and identification.

Four strains were isolated from the blood samples of three Chinese patients with candidemia and were named BMU05228, BMU05529, BMU05535, and BMU05314. Among those strains, BMU05529 and BMU05535 were isolated from the same patient at different infection stages. The patients were all male and were hospitalized in three different hospitals in China for treatment of colon, rectal, and lung cancer, respectively. The study was approved by the ethics committees of the involved hospitals. All the patients signed informed-consent forms. Four isolates were identified using sequencing of the nuclear ribosomal internal transcribed spacer (*ITS*) region and the large subunit of the 28S ribosomal DNA gene (*D1*/*D2*) as previously described (43, 44).

### Phenotype comparison of fungal strains.

**(i) Susceptibility testing.** The levels of *in vitro* susceptibility of these four strains to eight antifungal drugs were examined using Clinical and Laboratory Standards Institute (CLSI) broth microdilution method M27-A3. MICs of antifungals were interpreted according to CLSI document M27-S3 as described previously (10, 11). Susceptible and resistant phenotypes were determined according to methods described in previous reports (10, 11).

**(ii) Examining growth speed.** A 2,3-bis-(2-methoxy-4-nitro-5-sulfophenyl)-2H-tetrazolium-5-carboxanilide salt (XTT) colorimetric assay was performed for each C. haemulonii strain to compare the growth speeds at 30°C and 35°C. The cultures of each strain were supplemented with 100 μl XTT (1 mg/ml) containing 10 mM menadione at 0, 12, 24, 36, and 48 h after inoculation in 96-well plates, followed by an incubation at 37°C for 2 h under dark conditions. A 100-μl volume of supernatant was extracted, and the absorbance was examined by determination of the optical density at 490 nm (OD_490_).

**(iii) Observing the spore morphology.** The C. haemulonii isolates were inoculated in 10 ml yeast extract-peptone-dextrose (YPD) broth medium (Difco Laboratories, Sparks, MD) at 30°C and 200 rpm overnight. A 10-μl cell suspension was then placed on the slide and stained using 10 μl gossypol. The morphology of the spores was observed using light microscope.

**(iv) Examining stress fitness.** A spotting assay was used to examine the fitness of C. haemulonii isolates under conditions of CFW, SDS, and NaCl stress. Serially diluted fungal suspensions (2 μl) were inoculated onto YPD plates with different concentrations of CFW (15, 30, and 60 mg/liter), SDS (0.125%, 0.25%, and 0.5%), and NaCl (0.5 M, 1 M, and 1.5 M). The plates were incubated at 30°C for 48 h before observation.

**(v) Testing susceptibility to FK520 and polyoxin B.** A spotting assay was used to determine levels of susceptibility to FK520, an inhibitor of Cdr1, in C. haemulonii strains. Serially diluted fungal suspensions (2 μl) treated with 100 mg/liter FK520 were inoculated onto YPD plates with 64 mg/liter FLC. These plates were incubated at 30°C for 48 h before observation. Polyoxin B (DRE-E16283500; DRE, Germany) was used as an inhibitor of chitin synthase to examine the integrity of cell walls. The MIC value of polyoxin B for C. haemulonii was evaluated through methods similar to those described above.

### Genome sequencing, *de novo* assembly, and gene annotation of fungal strains.

Strains BMU05228 and BMU05314 were collected for genome DNA extraction after inoculation in YPD medium at 30°C and 200 rpm for 16 to 18 h. PCR-free Illumina MiSeq paired-end libraries with an insertion size of 400 bp were constructed using TruSeq DNA PCR-free library preparation kits (Illumina, USA). Each library was verified with respect to size with a model 2100 Bioanalyzer (Agilent Technologies, USA) and quantified according to the Illumina qPCR quantification protocol guide (Illumina, USA). Paired 250-bp ends were sequenced for each DNA fragment with an Illumina MiSeq sequencing platform. More than 75% of the reads achieved a phred quality score over 30 (45–47). Libraries of 20-kb SMRTbell templates were also prepared for BMU05228 and BMU05314 genome DNA. Libraries were checked for the quality and were quantified and sequenced using SMRT sequencing on a PacBio RS II system (Pacific Biosciences, USA). The raw polymerase reads were checked for the quality and were filtered using SMRT Analysis 2.3.0 (Pacific Biosciences, USA). Subreads were generated from polymerase reads for further analysis. Library preparation and sequencing and preprocessing of raw data were performed in Macrogen, South Korea. The preprocessed paired-end MiSeq reads and PacBio SMRT subreads were used for *de novo* assembly of BMU05228 and BMU05314 genomes both independently and jointly. SPAdes was used for MiSeq sequence assembly and for the hybrid assembly of MiSeq and PacBio sequences (47), while Canu was adopted for the assembly of PacBio sequences (48). To annotate the genes, for each strain, the RNA samples were collected from the fungi cultured under different conditions and at different stages and were pooled and sequenced. The details about RNA sequencing and data analysis are described in the following section. The transcriptome data were used for model training and prediction of gene frames with GeneMark (49). rRNAs were identified by alignment of known rRNAs against the fungal genomes, while tRNAs were identified with tRNAscan-SE (50). Peptide sequences were translated from the protein-encoding genes and aligned against the NCBI nonredundant (NR) protein database with BLAST. The homologous hits were further mapped to Gene Ontology (GO) Consortium or InterPro for functional annotation or family classification (51, 52). The proteins involved in pathogen-host interactions were downloaded from PHI-base and were also aligned against the proteome of BMU05228 or BMU05314 (53).

### Comparative genomic and phylogenomic analysis.

The genomes and encoded proteomes of representative strains of *Ascomycota* were downloaded from the NCBI Genome database. A bottom-up strategy was adopted to enable genome comparisons between the BMU05228 and BMU05314 strains. Briefly, prior knowledge about the phylogenetic relationships among the strains was obtained from the NCBI taxonomy database and literature, and a prior phylogenetic tree was constructed roughly. The two strains with the smallest phylogenetic distance were compared in a pairwise manner with respect to their proteomes, and the core proteome was determined. The core proteome was further compared with the proteome of the second closest strain or node with a determined core proteome. With this strategy, the core proteome (or genome) of *Ascomycota* was able to be determined at the final step. To reduce possible bias or complexity caused by paralogs, before the bottom-up core proteome analysis, a redundancy-filtering step was performed to remove any protein with two or more homologs in the same strain. Therefore, the proteins in the final core proteome set were all singletons in each examined strain. Homology was defined as representing greater than 30% similarity for more than 70% of the whole length of either pairwise proteins; mutual best hits were considered to represent orthologous pairs. The core protein (or gene) sequences were retrieved from each strain and concatenated according to a fixed order. MEGA6 was used to implement multiple-sequence alignments and reconstruction of the phylogenetic tree for the *Ascomycota* strains (54). Custom scripts were developed to assist the analysis.

### Genome resequencing and mutation calling of multidrug-resistant strains.

The genome DNAs of two multidrug-resistant strains, BMU05529 and BMU05535, were also extracted for resequencing. Illumina HiSeq 2500 paired-end libraries with an insertion size of 350 bp were constructed. Paired 250-bp ends were sequenced for each DNA fragment (Novogene, China). In total, 4,379,372 and 4,699,308 reads were obtained for BMU05529 and BMU05535, respectively. After adapter removal, the cleaned reads were mapped to the BMU05228 reference genome with bowtie (55). SAMtools was used to call the substitutions and small insertions or deletions (56). The mutation sites were further located with respect to the genome and genes. If a mutation site was within the frame of a protein-encoding gene, the effect was classified as a “missense,” “nonsense,” “sense,” frameshift,” “translation start codon missing,” or “translation stop codon missing” effect. If a mutation site was outside the frames of known protein-encoding genes, the distance between the site and the closest corresponding downstream gene was determined, followed by classification of the mutation site as “promoter” for a distance of <1,500 bp or as “intergenic” otherwise. The functional annotations and enrichments for interesting gene sets were performed with the tools provided by at the GO website (http://geneontology.org/).

### RNA extraction and sequencing and expression validation.

C. haemulonii cells were grown in YPD broth medium at 30°C with shaking at 200 rpm. The cells were treated with FLC at the final concentration of 1 mg/liter and incubated for 15 h. The cells were harvested, and total RNA was extracted using TRIzol reagent (Invitrogen) and prepared for RNA sequencing (RNA-Seq). Likewise, the cells were treated with FLC at the final concentration of 16 mg/liter at 10 h postincubation and incubated for another 5 h. Total RNA was then extracted and prepared for RNA sequencing. Additionally, after 15 h of incubation, total RNA from the cells without FLC treatment was used for RNA sequencing. The RNA sequencing reads were mapped to either the BMU05228 (C. haemulonii strain) reference genome or the BMU05314 (C. duobushaemulonii strain) reference genome with bowtie (55). The splice junctions were identified with tophat (57). Cufflinks was used to distinguish the isoforms and to quantify and compare the expression levels of the genes/isoforms (58). Statistical comparisons of the results of the gene expression analyses were performed with the cuffdiff module integrated in Cufflinks (58). For expression validation, total RNA was prepared for relative real-time quantitative PCR (qRT-PCR). According to the manufacturer’s instructions provided for the ReverAid first-strand cDNA synthesis kit (Thermo Science), first-strand cDNA was amplified from 1 μg total RNA by the use of oligo(dT) primers. Primers for qRT-PCR were designed and synthesized. The relative expression levels of genes were detected with an ABI 7900 system (Applied Biosystems) using SYBR green reagent (Applied Biosystems). Each sample was assayed in triplicate.

### Transgenic analysis.

Upstream and downstream 1-kb nucleic acid fragments of the *CH_4347* encoding frame in strain BMU05228 were amplified by PCR and ligated to the ends of an *AMP-HPH* fusion accordingly with NEBuilder HiFi DNA assembly reaction mix (NEB, USA). The *AMP* and *HPH* gene fragments were amplified from pYes2 and pAN-7 plasmid (Invitrogen, USA), respectively. The ligation product was transformed into Escherichia coli competent cells. Colony PCR and subsequent plasmid extraction were performed to confirm the successful construction and transformation of the ligated vector. The linearized vector with *CH_4347* flanking nucleic acid fragments was further transformed into BMU05228 competent cells by the use of Frozen-EZ Yeast Transformation II (Zymo Research, Canada). Peptone-dextrose agar (PDA) plates with 100 μg/ml hygromycin B were used for screening of fungi successfully transformed with the recombinant plasmid, and colony PCR was performed to screen the fungi in which recombination had happened. PCR was performed to amplify the fragment spanning the *CH_4347* encoding frame, and the product was sequenced to validate the successful knockout of *CH_4347* in BMU05228.

### Statistical analyses.

For analysis of RNA-Seq data, Cufflinks was used for transcript identification and expression comparisons as described above. All the graphs and diagrams from qRT-PCR were generated using the GraphPad Prism 6.0 (GraphPad, San Diego, CA) software package. Data shown represent means ± standard deviations (SD). Student’s *t* tests were performed for the comparisons, and a *P* value of <0.05 was considered to represent significance.

### Data availability.

All genome sequences and assemblies have been deposited in NCBI under the following BioProject accession numbers: for C. haemulonii BMU05228, accession no. PRJNA541353; for C. duobushaemulonii BMU05314, accession no. PRJNA541794. The RNA-Seq data have been deposited at NCBI under BioProject accession no. PRJNA541803. The scripts specifically designed for the bioinformatics analysis portion of this research are available via the following link: http://www.szu-bioinf.org/fungi/scripts.
