# Supplementary figures and images for: Global Screening of Genomic and Transcriptomic Factors Associated with Phenotype Differences between Multidrug-Resistant and -Susceptible Candida haemulonii Strains
Source: mSystems. 2019 Dec 17;4(6):e00459-19. doi: 10.1128/mSystems.00459-19 (PMC6918027; doi:10.1128/mSystems.00459-19)

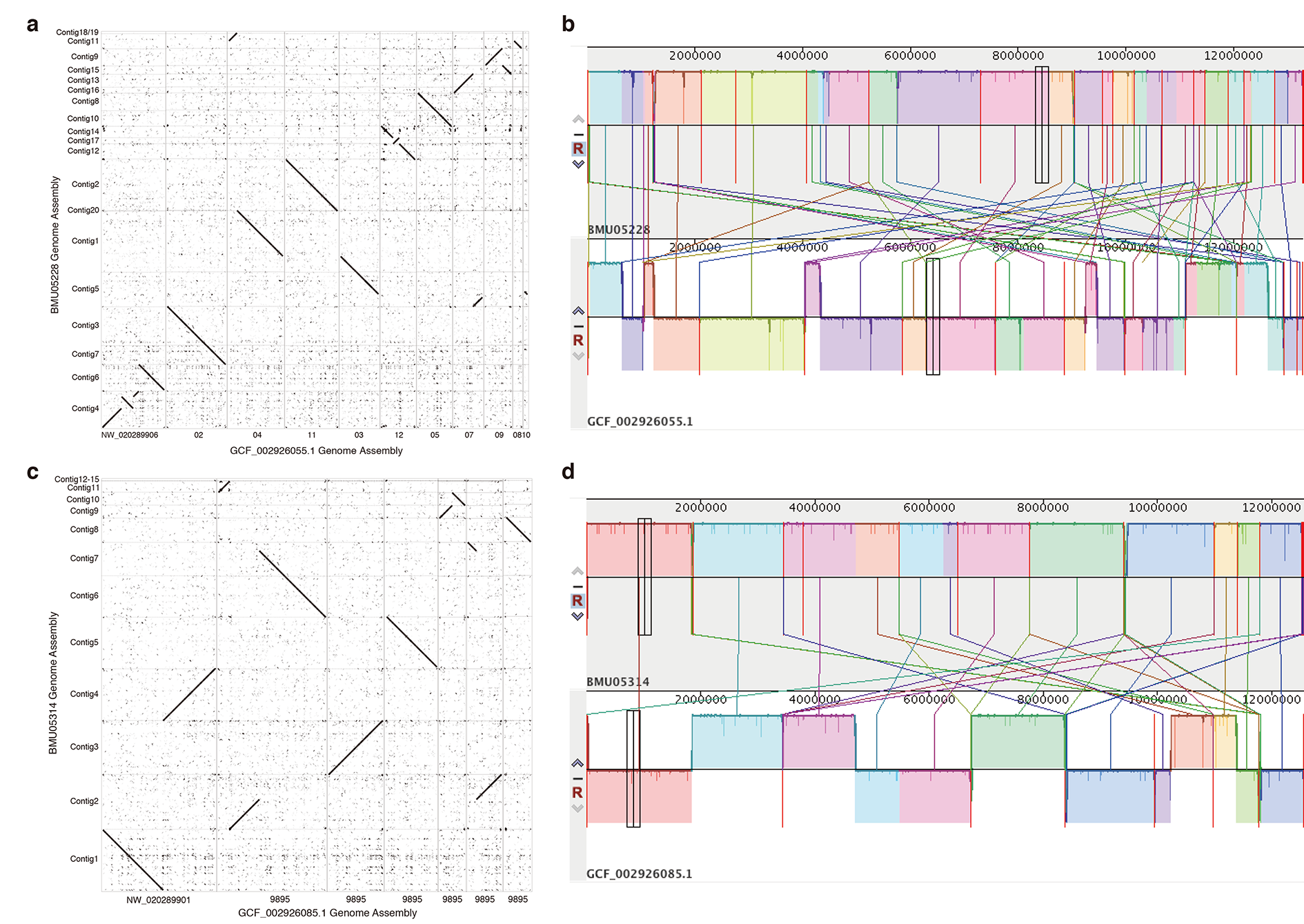

Supplement: FIG S1 [file mSystems.00459-19-sf001.tif]

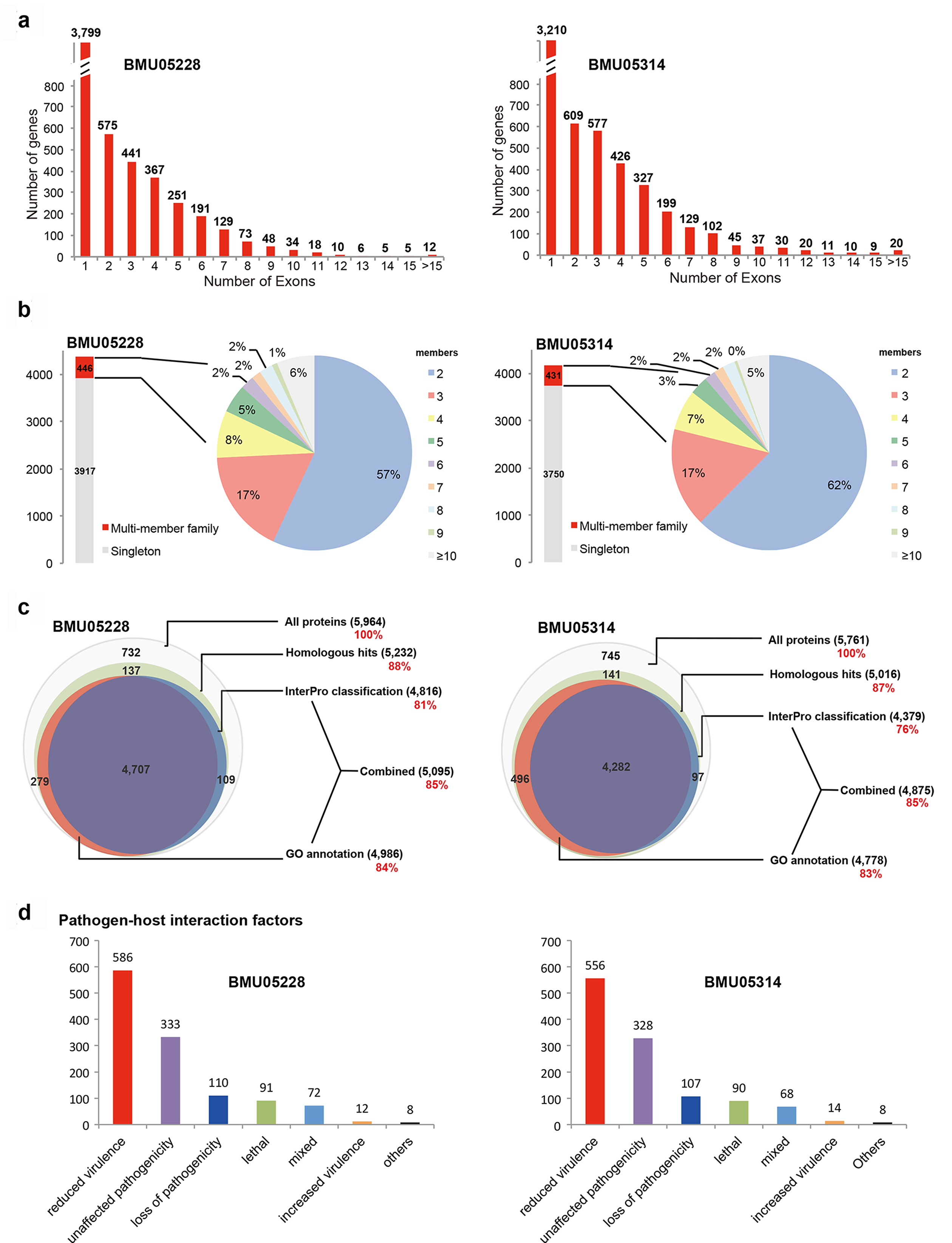

Supplement: FIG S2 [file mSystems.00459-19-sf002.tif]

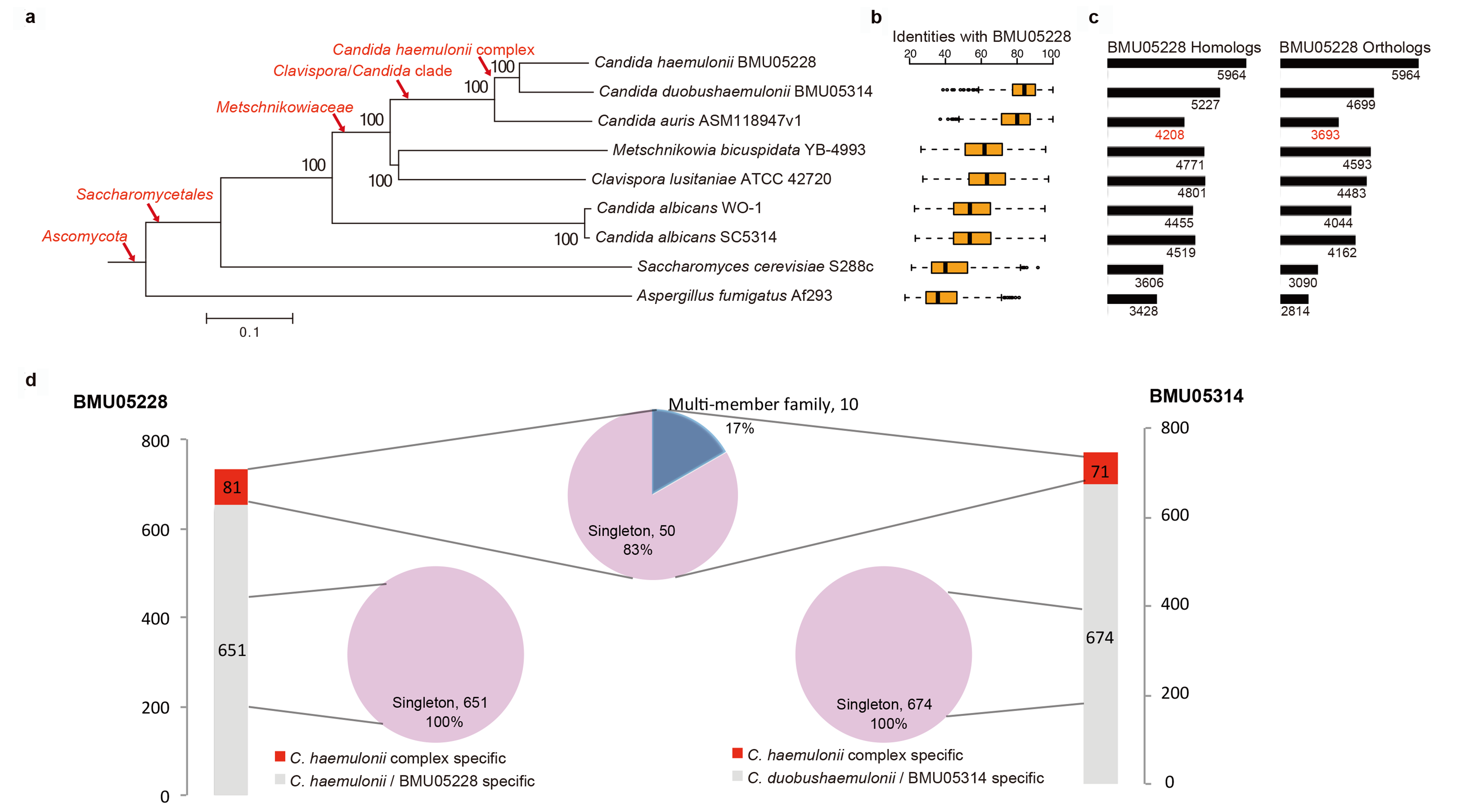

Supplement: FIG S3 [file mSystems.00459-19-sf003.tif]

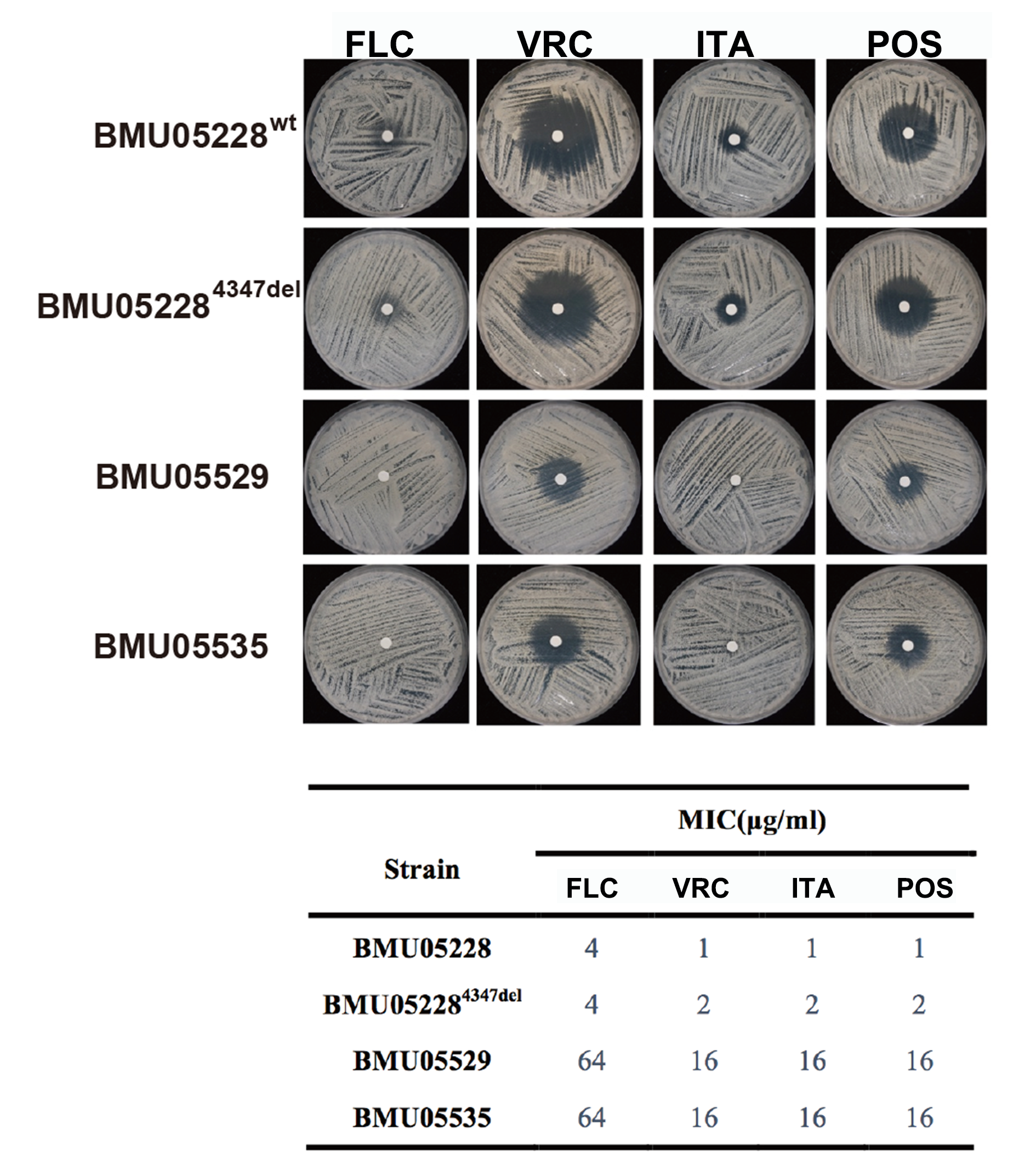

Supplement: FIG S4 [file mSystems.00459-19-sf004.tif]

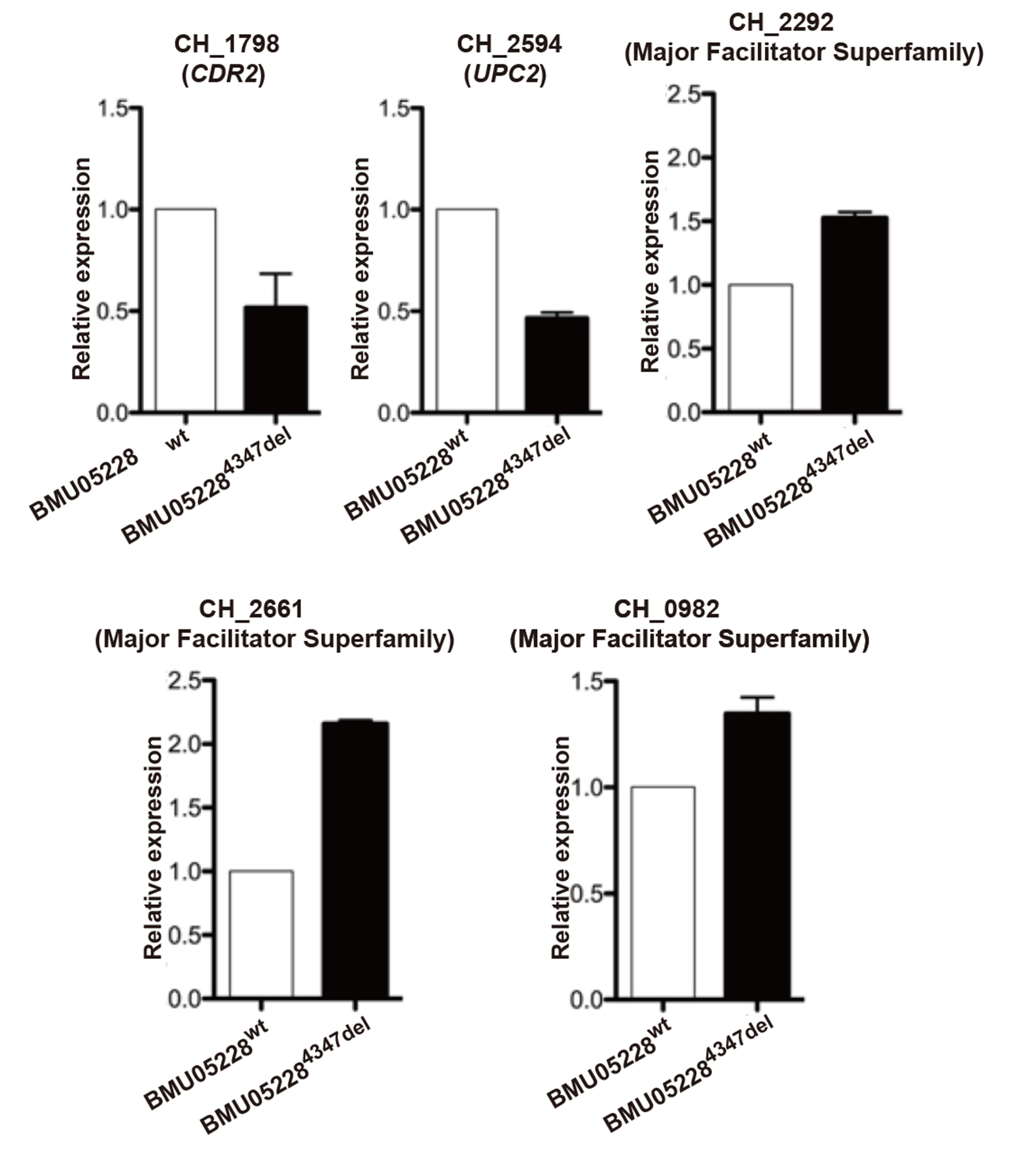

Supplement: FIG S5 [file mSystems.00459-19-sf005.tif]
