# Supplementary material for: Global Screening of Genomic and Transcriptomic Factors Associated with Phenotype Differences between Multidrug-Resistant and -Susceptible Candida haemulonii Strains
Source: mSystems. 2019 Dec 17;4(6):e00459-19. doi: 10.1128/mSystems.00459-19 (PMC6918027; doi:10.1128/mSystems.00459-19)
Supplement: TABLE S1 [file mSystems.00459-19-st001.docx]

**TABLE S1. Summary of genome sequencing and assemblies of *C. haemulonii* BMU05228 and *C. duobushaemulonii* BMU05314**

**Summary of genome sequencing**

| **Static** | **BMU05228** | **BMU05314** |
| --- | --- | --- |
| No. of Illumina MiSeq Reads | 16,289,658 | 13,109,128 |
| MiSeq Read Length | 250 bp | 250 bp |
| MiSeq Bases | 4,016,689,575 | 3,268,247,689 |
| No. of PacBio SMRT Subreads | 923,549 | 284,021 |
| Avg. Subread Length | 2,975 bp | 7,694 bp |
| Subread Bases | 2,747,433,231 | 2,185,317,181 |

**Summary of hybrid assembly**

| **Statistic** | **BMU05228** | **BMU05314** |
| --- | --- | --- |
| Total Length (nt) | 13,381,235 | 12,577,301 |
| No. of contigs | 187 | 51 |
| Largest contig (nt) | 2,059,638 | 1,868,465 |
| Contig N_50_ (nt) | 1,165,711 | 1,590,054 |
| Contig N_90_ (nt) | 428,779 | 741,123 |
| Contig N_99_ (nt) | 202,008 | 343,143 |
