# Supplementary material for: Global Screening of Genomic and Transcriptomic Factors Associated with Phenotype Differences between Multidrug-Resistant and -Susceptible Candida haemulonii Strains
Source: mSystems. 2019 Dec 17;4(6):e00459-19. doi: 10.1128/mSystems.00459-19 (PMC6918027; doi:10.1128/mSystems.00459-19)
Supplement: TABLE S2 [file mSystems.00459-19-st002.docx]

**TABLE S2. Genome summary and gene annotation of *C. haemulonii* strain BMU05228 and *C. duobushaemulonii* strain BMU05314**

| **Statistic** | **BMU05228** | **BMU05314** |
| --- | --- | --- |
| GC (%) | 45.19 | 46.85 |
| No. of total genes | 6,155 | 5,943 |
| No. of protein-encoding genes | 5,964 | 5,761 |
| Median protein-encoding gene length | 1,218 nt | 1,245 nt |
| No. of exons | 13,162 | 14,527 |
| Multi-exon genes | 2,165 | 2,551 |
| Single-exon genes | 3,799 | 3,210 |
| Median exon length | 209 nt | 151 nt |
| Proteins with alignment hits | 5,232 | 5,016 |
| Proteins with annotation | 5,095 | 4,875 |
| GO annotation | 4,986 | 4,778 |
| InterPro classification | 4,816 | 4,379 |
| Multi-member family | 446 | 431 |
| Single-member family | 3917 | 3750 |
| No. of rRNAs | 6 | 3 |
| No. of tRNAs | 185 | 179 |
